# The EEG as an index of neuromodulator balance in memory and mental illness

**DOI:** 10.3389/fnins.2014.00063

**Published:** 2014-04-08

**Authors:** Costa Vakalopoulos

**Affiliations:** Richmond Hill Medical CentreMelbourne, VIC, Australia

**Keywords:** EEG, memory, cholinergic, monoaminergic, REM, SWS

## Abstract

There is a strong correlation between signature EEG frequency patterns and the relative levels of distinct neuromodulators. These associations become particularly evident during the sleep-wake cycle. The monoamine-acetylcholine balance hypothesis is a theory of neurophysiological markers of the EEG and a detailed description of the findings that support this proposal are presented in this paper. According to this model alpha rhythm reflects the relative predominance of cholinergic muscarinic signals and delta rhythm that of monoaminergic receptor effects. Both high voltage synchronized rhythms are likely mediated by inhibitory Gαi/o-mediated transduction of inhibitory interneurons. Cognitively, alpha and delta EEG measures are proposed to indicate automatic and flexible strategies, respectively. Sleep is associated with marked changes in relative neuromodulator levels corresponding to EEG markers of distinct stages. Sleep studies on memory consolidation present some of the strongest evidence yet for the respective roles of monoaminergic and cholinergic projections in declarative and non-declarative memory processes, a key theoretical premise for understanding the data. Affective dysregulation is reflected in altered EEG patterns during sleep.

## Introduction

The EEG is one of the oldest non-invasive investigative tools of brain neurophysiology. It measures summed electrical currents generated by neural activity from multiple scalp electrode sites. Source localization remains poor, but the EEG measures changes in activity on a temporal scale unmatched by more modern imaging techniques, often in the range of only a few seconds. Progress has been made with the interpretation of EEG findings, but their significance in relation to cognitive function still remains relatively opaque. Renewed interest recently has been driven by the combination of EEG with functional imaging such as fMRI and PET scanning, which have poor temporal, but better spatial resolution and also by the opportunity of intracortical recording afforded by animal studies.

The present paper describes a theory of relative monoaminergic-cholinergic muscarinic balance (the MAB hypothesis) that underlies several well-described EEG rhythms. There are specific and interpretable qualitative and quantitative changes in the EEG that reflect cognition and its dysfunction. These can be explained, at least in part, by inverse neuromodulation of widespread cortical networks finely tuned to the effects of two main classes of neurotransmitter, acetylcholine (ACh) and the monoamines serotonin, dopamine, and noradrenaline. The section on sleep realizes the integration of the various threads of the theory of cholinergic modulation of cognition and affect. Sleep dysregulation is fundamental to biological mechanisms of depression. Imbalances of monoaminergic and cholinergic signaling are key attributes of the major sleep stages and these are reflected in and defined by EEG rhythms. The dissociation of memory consolidation during REM (rapid eye movement) and non REM (NREM) sleep provides compelling evidence for the dichotomy between implicit and explicit memory and in particular, their respective relationship to cholinergic and monoaminergic neuromodulation.

## EEG rhythms: cracking the code

### EEG alpha rhythms, the signature of the subconscious

The alpha rhythm of the electroencephalograph was originally described and is particularly dominant in a quietly resting state with eyes closed. The frequency band most commonly described in humans is between 8 and 12 Hz. It is generally thought that increased alpha activity signifies deactivation of the underlying cortex. Simultaneous EEG and functional magnetic resonance imaging (fMRI) recordings reveal a decreased magnetic resonance signal in the alpha state that involves multiple cortical areas (Goldman et al., 2002). These include occipital, superior temporal, inferior frontal, and cingulate cortex. Paradoxically, the insula activation positively correlates with the alpha rhythm suggesting a direct role in alpha generation. The link between alpha rhythm and cholinergic basal forebrain activity is thus, plausibly under the control of the insula. The insula, especially anterior agranular, is one of the few regions that project directly to cholinergic basal forebrain (Russchen et al., 1985; Grove, 1988). Insula activity as assessed on fMRI and PET scans is positively correlated with alpha power in both eyes closed and open conditions in normal subjects under passive conditions (Sadato et al., 1998; Goldman et al., 2002). These findings are of immense importance as most regions show reduced blood i.e., are negatively correlated with alpha power suggesting a source of generation of the rhythm.

Cholinergic muscarinic receptors are implicated in alpha rhythm generation. The muscarinic receptor antagonist scopolamine reduces both alpha power (Osipova et al., 2003) and coherence (Sloan et al., 1992). In amnesic patients with mild cognitive impairment alpha power was inversely proportional to estimated lesions of cortically projecting cholinergic white matter tracts (Babiloni et al., 2009). Although studies are sparse and inconsistent at least one study demonstrated reduced alpha power associated with the destruction of the nucleus Basalis (Riekkinen et al., 1990). Interestingly, increased delta power correlated with reduced choline acetyltransferase (ChAT) activity (see discussion later on delta).

Contrary to the idea that alpha oscillations are simply a reflection of resting states, the power of alpha is considered by others as a hallmark of sustained alertness and positively correlated with cingulo-insular-thalamic activity in one study (Sadaghiani et al., 2010). Higher per stimulus signal in this network linked to upper alpha band power improved perceptual performance for hits as compared to misses (Sadaghiani et al., 2009). In other studies reduced performance was accompanied by decreases in 10 Hz alpha power (Makeig and Inlow, 1993; Makeig and Jung, 1995).

A prominent idea is that alpha synchronization actively suppresses task irrelevant cortical activation both in intramodal and intermodal selective attention and working memory tasks. Physostigmine, a non-selective cholinergic agonist enhanced spatial attention by increasing alpha and beta activity in the hemisphere ipsilateral to the attended hemifield i.e., corresponding to the suppressed hemifield (Bauer et al., 2012). This pharmacological manipulation did not alter high frequency gamma oscillations, but did reduce reaction times. The authors however note that previous studies demonstrate an increase in cholinergic agonist-related visual cortex haemodynamic BOLD responses to attended stimuli (Furey et al., 2000; Bentley et al., 2003). Another study suggests that gamma frequencies can be obscured by alpha rhythms emanating from the same cortical topography and are revealed when studied as phase-locked events (Osipova et al., 2008). The results would confer a complex interpretation of EEG oscillatory activity and how it relates to neuromodulatory projecting systems and ultimately, both to performance. This is highlighted by another study, which showed physostigmine enhanced activity in the lateral orbitofrontal, anterior cingulate, temporal pole and left intraparietal sulcus to task-irrelevant fearful faces in “unattended” locations (Bentley et al., 2003).

The diversity and apparent contradictions of the findings can be explained by the projection of a network of cholinergic fibers acting on two different receptor subtypes M1 (excitatory) and M2 (inhibitory) on postsynaptic heteroreceptors. The receptors have opposing effects on cortical reactivity, but mediated by separate cortical neural substrates distributed in parallel. The prominence of a particular EEG spectrum of activity depends on not only the receptor subtype of a single class of receptor such as M1 Gαq/11 activating gamma rhythms and M2 Gαi/o inhibition and rhythmic rebound resulting in high voltage synchronization, but also on the relative levels of the two broad classes distinct neurotransmitters, of which the monoamines can antagonize muscarinic signal transductions. The MAB hypothesis determines the effects of varying levels of ACh vs. monoamines on EEG synchrony at particular frequencies and it's effects on cognition. Relative excess in the respective neurotransmitter molecule is proposed to result in either alpha or delta power increases. Synchronization is probably partly achieved through bursts of Gαi/o mediated inhibitory transduction of GABAergic interneurons and pyramidal cells (see **Appendix**). The actual frequency of synchronization in either the delta or alpha range would depend on unique signaling properties of monoamine and cholinergic muscarinic receptors, but both effect direct dampening of cortical activity related to unconscious and conscious processes, respectively.

The MAB hypothesis is modeled on a dual processing theory of cognition and the proposal for an inverse relationship between monoaminergic and cholinergic muscarinic receptor subtypes (Vakalopoulos, 2006, 2007). M1- and M2-type signaling are inversely modulated by 5-HT1A and 5-HT2A signals resulting in segregated couplings of 5-HT2A/C/M2 (conscious) and 5-HT1A/M1 (unconscious) receptor-modulated networks. This view suffices for the current purposes, but note that it doesn't exclude colocalization of Gαi/o with Gαq/11 in the same neurotransmitter class e.g., 5-HT2A/5-HT1A. The effector signal can be quite complex with synergies being established. Leaving this caveat aside, which could also apply to convergent signaling synergies of different neurotransmitter classes, a simple model of inverse modulation between ACh and a monoamine like serotonin or dopamine is most pertinent to the theory of generation of EEG rhythms proposed here. It will assist further in the interpretation of psychophysiological data.

**Figure 1:**
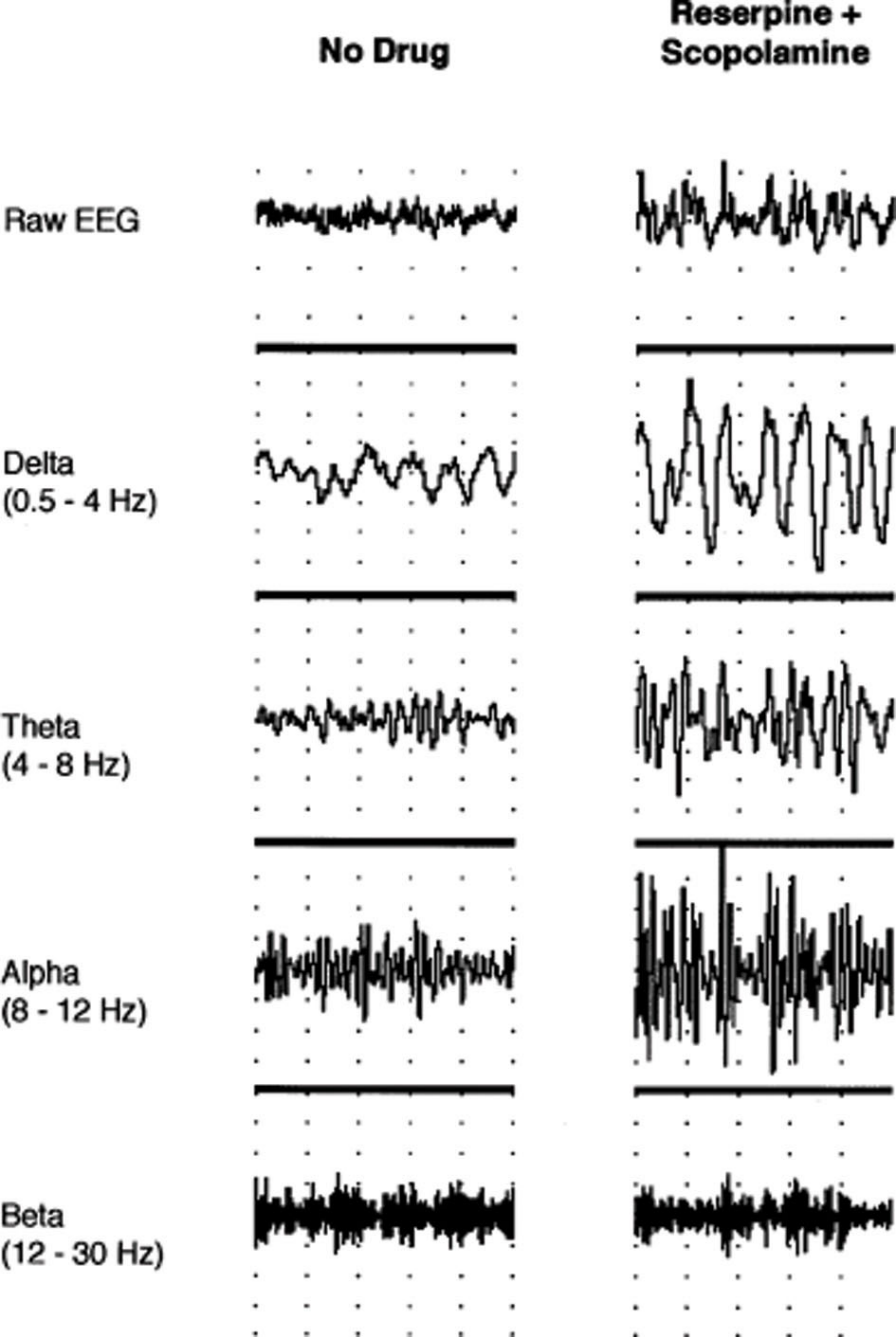
**Neocortical EEG activity in an awake, immobile rat prior to drug administration and after treatment with reserpine (10 mg/kg, i.p.) and scopolamine (1 mg/kg, i.p.).** Traces (from top to bottom) show raw EEG, delta (0.5–4 Hz), theta (4–8 Hz), alpha (8–12 Hz), and beta (12–30 Hz) activity, respectively. Note the increase in amplitude after reserpine and scopolamine treatment (1 s/division; 0.5 mV/division for raw EEG). Permission to reprint from Elsevier (Dringenberg and Diavolitsis, 2002).

While attention is momentarily engaged at one spatial locus or modality specific channel in a divided attention task, M2-mediated suppression of conscious information in unattended channels allows optimal performance, while still maintaining M1 receptor-mediated preattentive function for information of potentially high significance in the consciously suppressed channel. Functional inhibition of the 5-HT2A/2C-facilitated networks (serving here as the prototype receptor of monoaminergic systems) by M2 receptor would be reflected in observed alpha oscillations within the 8–12 Hz range in task-irrelevant areas as proposed by Jensen and Mazaheri (2010). A study where the administration of the selective serotonin reuptake inhibitor fluoxetine in reserpine-scopolamine treated rats, causing EEG slowing (Figure 1), restored EEG activation only when given concurrently with a 5-HT1A inhibitor (Dringenberg and Diavolitsis, 2002). Blocking raphe autoreceptors increases cortical serotonin levels and presumably through a post-synaptic 5-HT2A/2C mediated mechanism alters the balance with M2 muscarinic signaling. Under these conditions alpha synchrony was suppressed, but not delta, theta, or beta, suggesting alpha is the most sensitive marker of serotonergic action.

Fluoxetine also strongly potentiated EEG activation by tacrine, an acetylcholinesterase inhibitor (AChEI). Tacrine barely restores low voltage fast activity solely, that is, without a concomitant increase in serotonin levels. This is consistent with the idea that higher relative levels of ACh promotes alpha power. Tacrine does however suppress delta activity specifically (Dringenberg et al., 2000). In fact, tacrine increases alpha power at a dose that suppresses delta. Alpha is reduced to base levels by coadministration of the monoamine oxidase inhibitor pargyline. Tacrine at higher doses does suppress alpha power in a biphasic manner, but this may be mediated by direct antagonism of muscarinic receptors and other complex properties of the drug on neurotransmitter release and function that make it difficult to predict its effects (Becerra et al., 2001). The studies demonstrate a double dissociation of serotonin and ACh on EEG rhythms as predicted.

A question that remains unexplained is why a combined treatment that both lowers monoamines and non-selectively blocks the muscarinic receptor increases slow wave amplitude for both alpha and delta waves. The model predicts that the answer lies in the affinities of the muscarinic antagonist and monoamines at reduced levels of neurotransmitter that favors Gαi/o signaling for both classes of neurotransmitter.

### Conscious suppression as indexed by alpha

A number of experiments support the active suppression of conscious visual awareness in the context of alpha oscillations. Visual stimuli were presented at a threshold of 50% rate of detection while EEG recordings were taken (Busch et al., 2009). The study confirmed that stimuli preceded by strong alpha power were less likely to be perceived. In a metacontrast masking task, if the visual target coincides with the trough of the alpha cycle, subjects are less likely to detect the target (Mathewson et al., 2009). Reduced amplitude and delayed latency of the N1 event related potential also predicted undetected targets.

Alpha synchronization would indirectly index M1 activity predicted by a general rise in ACh level. N1 is a signal of preattentive processing and is modulated by cholinergic mechanisms presumably mediated by M1 receptor. Thus, higher synchronization in the alpha spectrum results in higher amplitude change in N1/N2 and P2 during an oddball task (Lee et al., 2011). The paradox of cholinergic-related alpha activation increasing detection of oddball or masked stimuli, is explained by a separately directed M1R-mediated alerting system or preattentive capacity for significant stimuli. The process can occur in the context of a simultaneous conscious suppression of non-attended information mediated by M2R. The alpha rhythm is a putative signal of M2R occupation. See **Appendix**.

### Event related potentials (ERPs) and the significance of N100 or N1

In a dichotic listening task the N1 component of the EEG was enhanced to all stimuli in the attended ear and showed a large deflection in the unattended ear (Hink et al., 1978). The P3 or P300 component was only present with attended target stimuli. N1 amplitude didn't predict P3 response in the attended ear suggesting discontinuous processes, but doesn't exclude the role of preattentive function directing or alerting subsequent attention and awareness to a significant event in both attended and unattended locations. The N1 component of an ERP is often regarded as the earliest top-down cortical response not to reach conscious awareness and reflects the initiation of a rapid response system. Conscious report involves a much longer time frame.

A number of studies implicate alpha synchronization in the generation of N1 component of the EEG. ERP average to non-target stimuli was large during the strongest post-stimulus alpha power (Makeig et al., 2002). The N1 is typically followed by “alpha ringing” in response to sudden visual stimuli. In a target auditory oddball paradigm larger N100's and shorter reaction times were associated with higher prestimulus alpha synchrony (Haig and Gordon, 1998). During a verbal recall to picture task, phase of alpha synchronization significantly impacts on N1 latency and generation of amplitude size (Gruber et al., 2005). Although, M2-type muscarinic activity is the proposed basis for alpha synchrony and thus remains inhibitory, alpha power indirectly indexes concomitant M1-type activity modulating networks that are parallel to those evoking alpha, but responding simultaneously to ACh release to help generate a N1 ERP signal.

Cholinergic manipulation has been shown to modulate the early ERP components. Rivastigmine, an AChEI, shortens N100 latency in novelty oddball tasks and biperidin, M1 muscarinic receptor antagonist, prolongs N100 and P200 latency during a paired-click task (Klinkenberg et al., 2012). Another muscarinic receptor antagonist, scopolamine delays N100 response to infrequent deviant tones (Pekkonen et al., 2005).

### Alpha memory

The left insula/Broca's area is one region where regional cerebral blood flow (rCBF) increases as demonstrated on PET during the identification of line drawings of animals or tools, but not when viewing nonsense objects (Martin et al., 1996). A decrease in alpha power was shown during picture recognition, there was none the less an increase in alpha phase synchronization when compared to meaningless objects (Freunberger et al., 2008). Semantic categorization is conceived as an automatic retrieval process of previously acquired knowledge. An earlier study also found that recognition of familiar objects induces a transient interhemispheric coherence of the alpha band, which does not occur with meaningless objects or their passive viewing (Mima et al., 2001). Importantly, the authors believed this to reflect the earliest stages of attention and recognition given the findings were already obvious from 117 to 373 ms. Alpha coherence during semantic access implicates an implicit process.

There is evidence for an association between alpha activity and familiarity-based judgments and routine behavior. An increase in both theta and alpha power in the rhinal cortex and hippocampus prior to word presentation predicted recognition performance (Fell et al., 2011). Rats foraging for food reward in a familiar environment demonstrate hippocampal and rhinal 10–12 Hz EEG rhythms, which disappear when in novel surrounds where an 8 Hz rhythm persists (Nerad and Bilkey, 2005).

### Alpha rhythms in affective disorders

A cholinergic theory of affective disorders states that prepotent behaviors associated with emotional dysregulation, such as impulsiveness, rumination, and inflexibility are encoded by implicit cortical traces that form networks with limbic areas (Vakalopoulos, 2007). The formation of unconscious memories that underlie motivation are proposed to be critically dependent on M1-type muscarinic receptor transduction involved in synaptic strength. Synaptic plasticity related to adaptation of behavior is a function of convergent monoaminergic actions and mediated by receptors including Gαi/o couplings e.g., 5-HT1A. Monoamine deficiency is a generally accepted model of depression and is explained here as a failure to antagonize cholinergic-mediated, but maladaptive prepotency of behavior. This paradigm can be applied to other conditions such as obsessive-compulsive disorder (OCD) or even ADHD. Alterations in the balance of neurotransmitter function across mental disorders could in principle, be indexed by EEG recordings.

In a subclinical cohort, a high urge to neutralize and obsessive compulsive-like thought was associated with increased alpha spectral activity in the left insula and ventrolateral prefrontal cortex during a negative visualization condition (Jones and Bhattacharya, 2012). There was an EEG correlation with the strongest feelings of guilt and anxiety. Subjects with poor sustained attention and who show difficulties in inhibiting distracting extraneous stimuli have a significantly larger proportion of alpha power in the lower frequency range (cited in Klimesch, 1999). These studies suggest a more complex role for alpha synchrony in approach behavior than the commonly held view of inhibition.

Depression severity shows a moderate positive correlation with left parieto-occipital upper alpha event-related synchronization during the maintenance period of a working memory task (Segrave et al., 2010). Patients displayed reduced accuracy. A negative correlation was observed in repetitive transcranial magnetic stimulation (rTMS) treatment-resistant depressive subjects between bilateral parieto-temporal alpha power and improvement in symptoms on a self-rated Beck Depression Inventory (BDI) score (Micoulaud-Franchi et al., 2012). Treatment responders to the selective serotonin reuptake inhibitor fluoxetine had greater initial alpha power than non-responders (Bruder et al., 2008). No change in alpha power was noted in either group after 12 weeks of treatment. Euthymic recovered elderly subjects maintained higher alpha amplitudes than controls and suggests spectral activity in this band may be a trait marker for depression susceptibility (Pollock and Schneider, 1989). However, another study showed that patients on 6 weeks of paroxetine had a diffuse reduction in alpha power with concomitant increases in delta, theta and beta power (Knott et al., 2002). Eighty percentage of subjects had more than a 50% reduction in Hamilton depression rating scale (HAM-D) scores. The latter study highlights a state related role of EEG alpha. This is supported by an early intrasubject study, in which there was an increase in alpha activity during the active phase of depression compared to the remission stage (Volavka et al., 1967).

A quantitative review of waking EEG data in depression, including unmedicated patients, concluded that the most consistent finding is elevated alpha power compared to controls (Pollock and Schneider, 1990). Not all studies are consistent (Price et al., 2008), but medication status and depression subtype i.e., bipolar disorder are generally confounded in many of these.

## Delta rhythms and volition

Recording EEG latencies to auditory tones revealed a functional, opposition between 10 Hz alpha and 4 Hz delta frequency systems as measured by a negative relationship of latency responses (Robinson, 1999). Whereas alpha power modulates early components of ERPs like N100, delta response dominates the P300 as shown by the analysis of an oddball paradigm (Demiralp et al., 2001). Awareness of perceptual switching in an apparent motion task was associated with an EEG wave that bore functional similarity to P300 and was prominent in the delta band (Başar-Eroglu et al., 1993). This component in a magnetoencephalography (MEG) study was interpreted as conscious awareness of change of multistable visual perception and destabilization prior to the switch was heralded by a decrease in alpha activity (Strüber and Herrmann, 2002). A study of voluntary control of Necker cube reversal showed larger gamma and delta responses with the attentional effort to slow the reversal rate (Mathes et al., 2006).

An outcome of the evolving model of inverse effects is the idea that synchrony in slow wave delta and faster alpha waves depend on the relative levels of monoaminergic and muscarinic cholinergic tone. EEG slow waves are an indirect measure of the significance of neuromodulator balance in the control of behavior. This proposal is partly built on a natural opposition demonstrated on certain task-related EEG activities analogous to that for the neuromodulators. Accordingly, delta would reflect increased tone of inhibitory monoamine receptor subtypes such as 5-HT1A and D2 on M1-modulated neuronal assemblies. An important prediction of the model is that delta power increases do not simply reflect suppression of learnt prepotent behaviors of subconscious origin, but index reversal of learning.

A related question arises as to what change in EEG signal is associated with stimulatory monoaminergic tone mediated through excitatory receptor subtypes? This can be gleaned from the study of reciprocal dynamics of EEG delta and alpha oscillations during spontaneous blinking. Delta blinking related oscillations are inversely related to alpha event-related synchronization and coincide with alpha event-related desynchronization and reduction in amplitude, ERD (Bonfiglio et al., 2011). ERD is a well-described phenomenon secondary to a putative increase in the ratio of convergent 5-HT2/D1:M2 signaling.

Attentional switching is associated with ERD of alpha in good performers, whereas inaccurate performers show hypersynchronization in a model of cognitive flexibility where the target alternated between a simultaneously presented digit and letter for each trial (Verstraeten and Cluydts, 2002). Good performers tended to show task-related desynchronization in the alternating protocol (difficult task) relative to same target response (easy task), whereas poor performers showed alpha synchronization irrespective difficulty of task. Significance was established relative to an open-eyes basal resting condition, but both groups showed attenuation of alpha power from eyes closed to open as expected. Desynchronization of alpha would represent better inhibitory control (or adaptability) rather than increased arousal, which authors proposed to explain their findings. Although not examined, higher concomitant delta power would be expected with good performance.

### A pharmacological model of EEG delta

**Figure 2:**
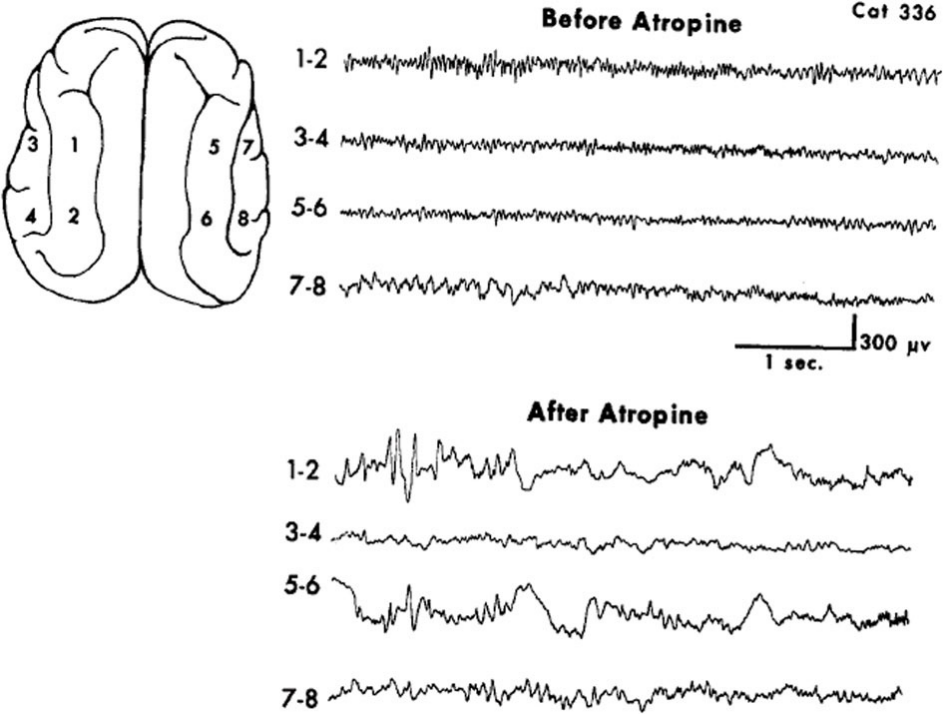
**Muscarinic blockade by the non-selective antagonist atropine elicits widespread slow oscillatory activity in the delta frequency range. From Schaul et al. (1978)**.

A limited number of studies explored the general pharmacological and more specifically receptor subtype effects on EEG patterns. These offer some support to the contention that the reciprocal nature of brain rhythms are embedded in the monoaminergic-muscarinic cholinergic dichotomy. The use of the AChEI rivastigmine in Alzheimer's disease decreases power in the delta and theta frequency bands and increases the lower alpha frequency band in the left insula for the subgroup manifesting cognitive improvement (Gianotti et al., 2008). A contradictory finding donepezil in mild Alzheimer's causes a magnitude increase in both delta and theta sources and conversely, a decrease in alpha (Babiloni et al., 2006). The apparent paradox is resolved by a study finding that in spite of being a weak AChEI donepezil exhibits anti-muscarinic activity unlike galantamine, another AChEI (Ago et al., 2011). This interpretation is supported by a study that found the appearance of EEG delta in cats treated with the general muscarinic antagonist atropine, Figure 2 (Schaul et al., 1978).

Use of ipsapirone, a 5-HT1A agonist, enhances slow wave activity during non-REM sleep in humans in the delta range with a corresponding dip at 11 Hz (Seifritz et al., 1996). The 5-HT2 receptor antagonist seganserin enhanced slow wave power during sleep, but unlike ipsapirone also augments alpha power, although non-significantly (Dijk et al., 1989). The opposing effects are predicted by a particular feature of the model as elaborated by Vakalopoulos (2006). Seganserin would alter the balance by enhancing M2 muscarinic cotransduction and thus, increasing alpha. The parallel increase in slow EEG rhythms may reflect the partial colocalization of 5-HT2 and 5-HT1A receptors and the blockade of the former would enhance the Gi/o signal inhibiting M1-modulated networks. The 5-HT2 receptors colocalized with M2, but not with 5-HT1A would represent functionally segregated networks. Compared to wild type mice, dopamine D2 knockout mice display a reduced power density in the lower delta frequency range during NREM sleep, at least during the dark cycle (Qu et al., 2010). The latter study extrapolates the findings to a more general model of monoaminergic effects on the EEG that includes dopamine, but may still demonstrate regional effects commensurate with innervation density.

### Delta and affective disorders

One of a small number of studies reveal a predicted generalized reduction in absolute power of the delta and beta bands in a group of patients with OCD where symptoms of depression were controlled for (Kuskowski et al., 1993). These changes were matched by widespread relative increases in alpha power. Findings of subsequent studies have been discrepant, although methodology and subgroups could explain some of the variability.

A promising tact would be to examine efficiency of delta response in these populations and the treatment response. One such study of major depressive disorder revealed an altered interaction between cardiac vagal influence and delta sleep (Jurysta et al., 2010). The authors claim that a measure of gain value reflects changes in neuroplasticity. In line with this suggestion an open label study of patients with major depression who demonstrated a good response to chronic therapy with the selective serotonin reuptake inhibitor paroxetine were examined for associated EEG changes (Knott et al., 2002). Increases in relative power of slow wave delta and theta and absolute power reduction in alpha band were noted.

## The alpha in ADHD: the EEG as a measure of imbalance of neuromodulator transduction

Hippocampal theta is associated with a phasic increase in the firing rate of most serotonergic neurons of the raphe and is a model of monoaminergic activation (Vakalopoulos, 2006). If theta predicts raised monoamine levels in the brain, then an inverse relationship with alpha should also be obvious in a normal population. In one study of typically developing children, alpha activity anticorrelated with mid-frontal theta (Mazaheri et al., 2010). A cue to expect a visual target reduced posterior alpha activity. None of these features were present in an ADHD comparison group between the ages 8–12. This is due to a putative deficient modulation of alpha by a top-down initiated dopamine D2-type receptors. It follows that impulsive or prepotent behavior can be indexed by higher alpha. In normal adults performing a Go/noGo task errors are predicted by prestimulus 10–11 Hz alpha and mu activity produced in occipital and sensorimotor regions, respectively (Mazaheri et al., 2009). It was suggested that central mu activity reflects an automatic response mode. Erroneous button presses induced increased frontal theta and a concomitant decrease in alpha.

During a serial reaction time task alpha power declined during initial learning and ERD was maximal when subjects acquired complete explicit knowledge of the sequence (Zhuang et al., 1997). Overlearning was associated with a decline in ERD and alpha synchronization. ERD and implicit learning are not mutually exclusive, however. Enhanced ERD is compatible with high tones in both monoaminergic and cholinergic systems. Once a sequence is well learned, increase in alpha power could result in theory, from either increasing relative cholinergic tone or its unmasking by reduced monoaminergic levels. As ERD declined RT continued to decrease. Thus, in a separate study hit rate and RT to near-threshold somatosensory stimuli was optimized at intermediate amplitudes of 10 Hz mu activity detected over sensorimotor cortex (Linkenkaer-Hansen et al., 2004). An inverted parabolic function best describes this putative measure of monoaminergic-cholinergic muscarinic balance for this task. In other words, a reduction or an increase beyond the optimal amplitude reduces performance and correlates with excess monoamine or acetylcholine transduction, respectively.

A theory of impulsive behavior and comorbidity in ADHD proposes higher relative cholinergic tone (Vakalopoulos, 2006). Supporting an interaction between monoaminergic and muscarinic signaling in ADHD two recent studies revealed up to a 50% reduction in muscarinic receptor binding in both lymphocytes and fibroblast cell homogenates (Coccini et al., 2009; Johansson et al., 2013). If this mirrors neuronal muscarinic receptor binding then it appears to be opposite to the expected direction. The real value of the EEG in the context of a reduction in absolute receptor numbers or their downregulation is relative tone associated with neuromodulatory balance. Thus, according to this model a finding of increase in alpha power reflects a reduced ratio of D2-type:M1 transduction irrespective of absolute receptor availability. The underlying aetiology of reduced muscarinic receptor numbers is presently unknown as is the reason for the presumed dopaminergic signaling deficit in ADHD.

Another finding inconsistent with the present model is the traditional belief of a general increase in theta power and theta/beta ratio and a decrease in alpha. A study looking at EEG defined subtypes conforms to this general impression with only the minority group demonstrating elevated alpha and reduced theta (Clarke et al., 2011). A conspicuous feature of the data was the age differential between the first 4 clusters demonstrating reduced global alpha power 9.4, 8.2, 8.9, and 8.3 years and the last small cluster where alpha was enhanced, 12 years. The range of ages of ADHD subjects recruited for these studies straddle major developmental phases. One study challenges the classical interpretation and use of fixed frequency bands citing the evolving maturity of peak alpha frequency begins at 4–6 Hz in the first year and increases to about 10 Hz by age 10 (Lansbergen et al., 2011). By using individualized frequency bands as first recommended by Klimesch (1999) they found that a significant proportion of the variation ascribed to theta can be subsumed under slow alpha peak frequencies. In these boys with ADHD impaired performance in the Go/noGo task positively correlated with the alpha1 frequency band.

An excess of theta extended to an adolescent group with ADHD compared to controls, but there was a parallel increase in alpha1 (8–9 Hz) (Lazzaro et al., 1999). Absolute alpha activity was larger in frontal and midline sites and there was no increase in absolute delta. Children with ADHD between the ages 6–16.1 and a mean of 10.8 showed an excess of relative alpha in addition to absolute theta power and a decreased alpha and beta mean frequency especially in frontal regions, a major site of dopamine projections (Chabot et al., 1999). A generalized decrease in absolute and relative delta power was another feature of the study and conforms to the proposed model of reduced dopamine D2-type tone. The alpha excess replicated an earlier study by the same group (Chabot and Serfontein, 1996). The general findings for delta activity are not always consistent, but the children who benefitted most from stimulant therapy had an excess of alpha and beta relative power. A recent study showed higher alpha levels upon opening eyes condition relative to closed in children with ADHD that is, less desynchronization than controls (Fonseca et al., 2013). This was prominent in frontal regions consistent with higher dopamine innervation. Consistent with the idea that impaired dopamine signaling increases alpha is a study of chemically induced parkinsonism in monkeys inducing the appearance of global alpha rhythm at 10 Hz (Goldberg et al., 2004).

Girls with ADHD compared to controls had reduced midline absolute delta and elevated alpha as predicted (Dupuy et al., 2013). The mean age was 10 years and the study used a standard frequency value range. This differs from the findings on boys by the same group and may reflect maturational differences between the sexes. The combined typed also had lower relative in the right hemisphere and more absolute alpha in the temporal-posterior region compared to the inattentive type. This is consistent with the prediction of a selective D2-type signaling deficiency associated with hyperactive-impulsive dimension and D1-type deficit associated with inattention (Vakalopoulos, 2007).

Heterogeneity within the disorder classed as ADHD suggests a variation in EEG findings as comorbid bipolar spectrum may be expected not to present the same neurophysiological anomalies as a more homogeneous “core” group. Note also that the prediction of the model assumes impaired dopamine signal transduction, but doesn't address the role of serotonergic modulation of alpha rhythms, which provides a theoretical confound for the neurophysiological data. That is, does 5-HT1A neuromodulation compensate for aberrant D2/4 dopamine receptor signal as measured by EEG alpha or could polymorphisms and altered function of the former receptor subtype result in a distinct subgroup of ADHD with overlapping clinical features?

## Sleep, the EEG, and the monoamine-acetylcholine balance (MAB) hypothesis

Sleep stages are segregated into a number of neurophysiological and neurochemical events and broadly divided into non-REM, including slow wave sleep (SWS), and REM or paradoxical sleep stages. SWS is characterized by large amplitude slow frequency waves in the delta range i.e., 0.5–4 Hz. A unique feature of SWS is ACh levels in the forebrain are at a minimum (Poe et al., 2010). Compared to waking there is a reduced but sustained availability of noradrenaline and serotonin (Gais et al., 2011). A muscarinic M1 partial agonist increases wake periods and reduces SWS in rats (Iwata et al., 2000). In anaesthetized monkeys the same agent increased alpha and beta power, but also reduced the dominance of delta activity. During REM sleep there is a reciprocal increase in the cortex and hippocampus of ACh (Marrosu et al., 1995), while serotonin and noradrenaline levels are at a minimum (Aston-Jones and Bloom, 1981; Park et al., 1999). An EEG signature of REM is desynchronized activity, but alpha remains an important spectral power component (Cantero et al., 2000) and is generally slower (Gelisse and Crespel, 2008). Paradoxical refers to the desynchronized activity normally indicative of an alert state. This can be attributed to the concurrent activation of dopaminergic systems, which do not appear to vary during sleep stages. Real time sleep analysis demonstrates reduction of alpha activity during the transition from wake to sleep and is at a minimum during stages 3 and 4 (Kuwahara et al., 1988). EEG rhythms during sleep present a unique opportunity to study neurochemical associations of cognition.

### REM and SWS have specific effects on memory consolidation

A study reported the differential effects of early and late sleep stages on consolidation of declarative and procedural memory tasks (Plihal and Born, 1997). Explicit recall of paired-associate lists benefitted most from SWS and mirror-tracing skills from REM sleep. The seminal study demonstrating specific SWS consolidation of paired associates was Yaroush et al. (1971). Low cholinergic tone is critical to postlearning consolidation of declarative memories during SWS. Infusion of the cholinesterase inhibitor physostigmine completely blocked SWS-related consolidation of word pairs in human subjects, but did not interfere with the consolidation of mirror tracing, a procedural task (Gais and Born, 2004). 30 min post-training administration of combined muscarinic and nicotinic cholinergic antagonists scopolamine and mecamylamine improved consolidation of word pairs (Rasch et al., 2006). Most explanations of the paradox of low cholinergic tone facilitating declarative memory consolidation appear *ad hoc* at best. The MAB hypothesis states that monoaminergic tone is enhanced by low cholinergic activity.

The prediction is based on an application of the theory of hippocampal-dependent phasic activation of monoaminergic projections facilitating synaptic connections in activated cortical networks for the specific acquisition of declarative memories (Vakalopoulos, 2006). Although, SWS has been viewed as a hypometabolic state there is a generalized and selective increase in protein synthesis during this stage (Ramm and Smith, 1990). During SWS the process of encoding declarative memory traces is already advanced and consolidation is robust in the context of a hypocholinergic state. Compare this to initial encoding, which observes an inverted U-type function of optimal monoaminergic transduction levels and is acutely sensitive to muscarinic cholinergic blockade. The latter may contribute to the relative paucity of subsequent dream recall from SWS. An explanation of the dissociation between procedural consolidation and reduced dream recall in REM sleep is more straightforward considering a relative hypoaminergic state.

Recall of dreams in late predominantly REM sleep was associated with significant increases in delta amplitude in the transition between sleep and awake as compared to failed recall in an intersubject study (Rochlen et al., 1998). If delta amplitude is an index of a raised MAB ratio then this supports the hypothesis of elevated phasic monoamine levels being necessary for explicit recall. The difference in delta magnitude was greater for depressed patients than controls during successful recall. Recall failure was characterized by small EEG shifts from sleep to wakefulness in the depressed group, but not healthy controls consistent with a dysfunction of monoamine transduction in depression. Dream recall rates are extremely low in depression (Armitage et al., 1995). In those subjects treated with 2–4 weeks of antidepressants overall recall rates were even lower especially for nefazodone, a 5-HT2 antagonist (Rochlen et al., 1998). Just under a third of those treated with fluoxetine were the exceptions showing increased dream recall. According to the model, 5-HT2 blockers would impair explicit recall, whereas raised levels of serotonin with fluoxetine would positively modulate 5-HT2A/CR.

A short daytime nap consisting exclusively of NREM sleep, 48% SWS, improves paired associate learning, but not mirror tracing (Tucker et al., 2006). Improvement in a visual discrimination task, which relies on preattentive capacity, positively correlated with REM sleep, but not SWS during a nap (Mednick et al., 2003). Transition from implicit knowledge of hidden task regularities to explicit insight of a number reduction task was especially associated with SWS and this was irrespective of whether implicit knowledge was evident during learning (Yordanova et al., 2011). Delta power in SWS is a direct index of MAB, but a putative indirect measure of explicit consolidation, which occurs in networks exhibiting low-voltage fast activity (LVFA) in the gamma range. LVFA is frequently seen background to SWS periods (Destexhe et al., 1999).

### Hippocampus encodes implicit spatial memories

A major confound in the literature is the assumed strict equivalence between hippocampal-dependent and declarative memories. Direct encoding of allocentric spatial relations, both implicit and explicit in origin, occur within the substrate of the hippocampus itself (see discussion in Vakalopoulos, 2006). This is separate to its putative role in phasic activation of brainstem nuclei that facilitate encoding of declarative memories in the cortex and is related to theta hippocampal rhythm by raising monoaminergic levels. Thus, damage to the structure with overlapping roles can misattribute findings to a single memory system. This is especially true of animal studies where differentiating which strategy is used to perform spatial tasks is difficult.

One of the most prominent theories of declarative memory consolidation is the transfer of information from the hippocampus to the neocortex during sleep. A study contradicts this supposition suggesting direct encoding of a memory trace with immediate early gene up- regulation in the prelimbic region (PrL) of the rat cortex during learning of an animal model of the paired associate task (Tse et al., 2011). Direct injection of glutamate receptor antagonists into PrL disrupted learning of paired odor-location. Increased relative monoamine levels triggered by hippocampal theta activation and during SWS is a more parsimonious explanation of encoding and consolidation.

### REM sleep and procedural memory

Consolidation of procedural memory is generally believed to be a function of REM sleep. REM but not non-REM sleep deprivation resulted in a lack of performance gain in a preattentive visual discrimination task (Karni et al., 1994). In a sequential motor task sleep enhanced speed of performance by a third and reduced the error rate by 30% on average (Fischer et al., 2002). The gains were positively correlated with high amounts of REM sleep. Specificity of consolidation during relative REM cholinergic excess is demonstrated by a study, which shows regional cerebral reactivation of the cuneus only after implicit probabilistic learning in a serial reaction time task, but not to random task-related visuomotor improvements (Peigneux et al., 2003).

Rats trained on inhibitory avoidance (IA) and cued and contextual fear conditioning tasks after 96 h of REM sleep deprivation (REMD) showed decrements in performance as measured by latency and freezing time (Dametto et al., 2002). The authors favored an encoding deficit as an explanation. That cholinergic systems are involved was suggested by a study showing reversal of IA deficit when pilocarpine, but not atropine was administered during REMD (Bueno et al., 2000). REMD desensitizes post-synaptic muscarinic receptors (Tufik et al., 1987), a proposal supported by study demonstrating reduced M2 receptor binding (Nunes et al., 1994). Post training infusion of the muscarinic cholinergic agonist oxotremorine into the basolateral amygdala (BLA) in rats enhances contextual memory and the same agent infused into the dorsal hippocampus enhanced retention for IA (see Power et al., 2003). The selective M1 receptor antagonist dicyclomine impaired both contextual fear conditioning and IA, but not tone fear conditioning when given prior to acquisition training (Fornari et al., 2000). Cued fear conditioning may involve a distinct muscarinic receptor subtype since pretraining use of scopolamine impairs both contextual and auditory cued-fear conditioning (Rudy, 1996).

Scopolamine also impaired consolidation of both types of fear conditioning when administered up to 3 h post training. However, for tone-cued conditioning this was evident only after a single initial pairing of auditory cue and shock, but not when rats received 3 pairings. Rapid overlearning may thus occur for the cued task. By analogy, sleep facilitated consolidation of contextual fear, but not cued fear in mice exposed to 3 tone-shock pairings (Cai et al., 2009). The authors interpreted the distinct effects as a consequence of hippocampal-dependent vs. independent tasks. However, a lower threshold for ceiling effects in the cued task offers an alternative explanation for one of essentially two implicitly acquired tasks. Paradoxical sleep presents an ideal neurochemical milieu for consolidation of implicit memories as low monoaminergic tone would enhance cholinergic transduction.

### The substrates of implicit learning as revealed by paroxysmal sleep (PS or REM)

Fear conditioning to a tone paired with electric shock in awake rats induced an enhanced response in the lateral amygdala and medial part of the medial geniculate nucleus (MGm), a thalamic relay of auditory information (Hennevin et al., 1993, 1998). The conditioned response to the tone was maintained during REM, but not non-REM sleep in MGm and primary auditory cortex (Hennevin and Maho, 2005). MGm projects directly to the striatum, a feature of the intralaminar thalamus, and to the amygdala (Moriizumi and Hattori, 1992). In an intriguing study of a two-way active avoidance shuttle box, retention and subsequent performance in a session by rats was better when the conditioned stimulus was presented during intervening PS than during waking or no stimulus conditions (Hars et al., 1985).

Conversely, the medial pulvinar (mPul), part of the classic thalamus, is uniquely dissociated from cortical activity during paroxysmal sleep (Magnin et al., 2004). This relative functional decoupling from only this vigilance state corresponds to an extremely weak cholinergic innervation of mPul (Hirai and Jones, 1989) compared to high levels of cortical cholinergic activation associated with PS. The mPul is inclusive of a parallel thalamocortical network that figures prominently in consciousness theory and contrasts with the putative role of the intralaminar group.

That implicit learning implicates the intralaminar nuclei was predicted by Vakalopoulos (2005) as was the importance of the relative muscarinic activation, which remains high during REM, but is lowest in non-REM sleep. The model further predicts an exception of enhancement of activity by the conditioned tone during periods of alpha intrusion into SWS. Innervation of the thalamus by the basal forebrain using discrete deposits of anterograde tracer is restricted to the intralaminar, midline and mediodorsal nuclei (Kolmac and Mitrofanis, 1999). This suggests a highly selective association of cholinergic cortical activation with the thalamus, although no controls for spread of tracer to surrounding structures were made. Stimulation of the parafascicular nucleus attenuated the deficits in active avoidance associated with lesions of the cholinergic nucleus basalis magnocellularis (Sos-Hinojosa et al., 2003).

### Sleep parameters surrounding flexible behavior

The Morris water maze is a classic hippocampal-dependent animal experiment that is considered to be homologous to human declarative memory. It involves finding a submerged hidden platform in a fixed quadrant of a water tank using extraneous cues. However, it is plausible that this task can be solved using a procedural strategy and still retain a dependence on hippocampal integrity because of an implicit allocentric representation encoded in this structure. REMD in a recent study impaired initial task acquisition, but only after a four trials per day protocol (incomplete learning). This was assessed by a probe test of time spent in the target quadrant when the platform was removed (Walsh et al., 2011). By contrast the REMD group performed better than controls on a measure of proximity to platform during the probe for the location reversal phase of the task. REMD and a relative hypocholinergic state would interfere with implicit consolidation of the original platform location, thus making it easier to learn a new location. Intact non-REM and SWS in particular, would facilitate reversal.

A separate study involved visuomotor learning where subjects reach for targets using a cursor while adapting to covert systematic rotations of the perceived cursor path (Huber et al., 2004). The authors view the task as an implicit learning paradigm, but error detection and correction are largely guided explicitly. Pertaining to the complexity of the task subjects were required to adapt to four incremental steps of 15°. They found a specific increase in SWS activity in the 1–4 Hz range localized to the right parietal cortex and which correlated positively with reduced directional error. In summary SWS is a neurophysiological marker of adaptive behavior related to relative monoamine neuromodulatory excess.

### Orexins

Circadian rhythm can be manifestly disrupted in depressive illness. A combination of orexin receptor 1 and 2 antagonists can reduce latency to REM and increase duration at the expense of NREM sleep (Dugovic et al., 2014). The effect of OXR2R antagonist alone reduced latency of NREM sleep and its duration. Orexin antagonists have been considered as potential treatments for various disorders including addiction, depression and anxiety (Pich and Melotto, 2014; Yeoh et al., 2014). The orexinergic neurons of the lateral hypothalamus modulate monoaminergic and cholinergic projections implied in cortical arousal (Saper et al., 2001). Thus, the effects of orexin on sleep dysfunction in affective disorders could be mediated by differential effects on monoamine-cholinergic imbalance and these in turn help determine the changes in EEG profile.

## Sleep EEG in affective disorders

### Sleep related memory consolidation in children and ADHD

Children normally outperform adults in declarative memory consolidation after sleep as demonstrated by explicit post sleep knowledge gains of an implicitly acquired motor sequence (Wilhelm et al., 2013). In both children and adults the transition showed a significant association with SWS. SWS was three fold higher in children than adults, a generally well documented finding (Ohayon et al., 2004). Post sleep retention of word pairs correlated positively with non-REM, but negatively with REM sleep (Backhaus et al., 2008). The children in this study spent more than 40% of total sleep time as SWS. Strikingly, children show a parallel deficit in consolidation of procedural memory during sleep as revealed by a serial reaction time task (Fischer et al., 2007). The deterioration in reaction time (RT) differences between grammatical and non-grammatical trials was specific to the retention interval as the rate of improvement during initial learning was greater in children, albeit slower than adults overall. It appears that better explicit memory consolidation occurs at the expense of implicit retention. Compared to adults, children exhibited an increase in SWS, but similar levels of REM.

Putative dysfunction of components of the monoaminergic system in ADHD would be expected to signal specific changes in neurophysiology and cognition during sleep. From the limited number of studies available it appears that there is an inverse relationship to normal findings in children. Deficits in declarative consolidation would be expected, but what is particularly revealing is the enhanced adult-like procedural improvements. Qualitative changes in SWS are apparent (Wilhelm et al., 2012) and these changes may underlie the impaired consolidation of a picture recognition task by subjects with ADHD as compared to normal children (Prehn-Kristensen et al., 2011a). The task can be solved implicitly, but a protocol requiring subjects to judge the level of arousal during the encoding phase of each picture should facilitate a declarative strategy. Importantly, memory consolidation showed a positive correlation with delta and slow oscillation power in controls but not in subjects with ADHD. Performance was comparable in the wake condition. Slow oscillation power did not differ between groups, but ADHD subjects did display significantly prolonged REM sleep compared to controls. Contrary to typically developing children and consistent with the REM findings, ADHD subjects benefitted from the sleep consolidation or normalization of procedural learning in a serial reaction time task (Prehn-Kristensen et al., 2011b). RT gain was positively correlated with percentage of S4 stage and REM sleep and is compatible with a 2-stage model of memory consolidation. SWS would facilitate flexible organization of learned sequences and REM sleep selectively unmasks potentiated cholinergic modulation in children with ADHD as predicted by the MAB hypothesis. Acquisition of a motor task clearly solicits implicit and explicit strategies (Cleeremans, 2008).

### Sleep related EEG changes in the treatment of depression

Decreases in duration of SWS and amplitude of delta power density are robust findings in unmedicated depressive patients (Borbély et al., 1984; Gillin and Borbély, 1985). Patients treated with clomipramine, a tricyclic antidepressant, showed a correlation of clinical response with an increase in delta power (Kupfer et al., 1989). A study of rTMS in cases of resistant depression to pharmacotherapy measured alpha rhythm during REM sleep (Pelliciari et al., 2013). Reduction in symptoms correlated with decreased REM alpha power in the dorsolateral prefrontal cortex (DLPFC). There was no change in REM macrostructure. Another preliminary open-label study found an increase in SWS delta power, at least in the initial course of treatment with DLPFC rTMS (Saeki et al., 2013). The authors of the former study suggested that symptom improvement was a function of increased cortical activity since alpha was an inhibitory measure, but also noticed the limitations of such an interpretation. More likely, the EEG changes of the two studies reflect a change in the balance of transduction efficiency of monoaminergic relative to cholinergic muscarinic transduction. A link between waking synaptic plasticity and sleep slow wave activity was found in a study of limb immobilization that resulted in reduced cortical somatosensory evoked potentials and performance decrements in a reaching for target task (Huber et al., 2006).

## Conclusion

It has long been regarded that the transition from slow-wave synchronization to LVFA represents a state of change in arousal. Early studies implicated a reticular activating system that current evidence attributes to activity within monoaminergic and cholinergic nuclei. However, more recent studies and the distinct neurochemical nature of sleep stages reveal a rather more complex relationship between high-voltage synchronized activity and cognitive function, including memory consolidation. The paper offers a model for interpreting several EEG rhythms based on relative neuromodulator levels and the associated putative implicit and explicit psychological constructs.

### Conflict of interest statement

The author declares that the research was conducted in the absence of any commercial or financial relationships that could be construed as a potential conflict of interest.

## Appendix

Rhythmic fluctuations of membrane potential (Vm) in the delta frequency ranges (0.5–4 Hz) in urethane-anaesthetized rats model naturally occurring SWS (Metherate and Ashe, 1993). Long-lasting hyperpolarizations are mediated by K^+^ influx beyond the typical Cl^−^ related IPSP (inhibitory post-synaptic potential). Thalamocortical projections are not necessary for cortical delta rhythms (Timofeev and Chauvette, 2011). Gi/o suppression of GABAergic interneurons and pyramidal neurons can result in rebound synchronous depolarizations. The ubiquitous presence of Gi/o-coupled GIRKs (G-protein activated inwardly rectifying K^+^ currents) could underlie rhythmic slow high-amplitude oscillations. GIRKs are activated by the Gβγ subunit (Ivanina et al., 2004). D2-dopamine and 5-HT1A receptors activate Gi/o and GIRKs (Wiens et al., 1998; Jaén and Doupnik, 2005; Webb et al., 2005). In the urethane rat model of delta rhythms stimulation of the cholinergic nucleus basalis causes cortical activation through muscarinic blockade of inwardly rectifying currents, K_ir_ (Metherate and Ashe, 1993). M1R activation of Gq and phospholipase C (PLC) desensitizes GIRK currents (Kobrinsky et al., 2000) antagonizing monoamine-dependant delta generation.

Gαi activation of GIRK also responds to M2R (Ivanina et al., 2004). K^+^ current induced hyperpolarizations could plausibly serve generation of alpha oscillations. The higher frequency range of EEG alpha clearly entails factors that alter membrane kinetics. A source of rapid desensitization of K^+^ currents is the regulator of G-protein signaling (RGS4), which markedly accelerates GIRK deactivation (Doupnik et al., 1997). M1R colocalization in a proportion of M2 expressing neurons suggests a role in Gq-mediated desensitization of GIRK (Kobrinsky et al., 2000). Hydrolysis of phosphatidyl inositol biphosphate (PIP_2_) causes sequestration of Gβγ and thus, termination of GIRK currents. In the absence of RGS proteins serotonin exhibited a 30-fold increase in GIRK activity at 5-HT1AR than ACh at M2R (Jaén and Doupnik, 2005), showing differences in affinity that may be important for frequency selection. Thus, M1 would desynchronize 5-HT1A-induced delta (M1 dominant), but shorten M2-related oscillations to an alpha frequency (M1 subservient). The idea is that there are segregated intracortical sources of ACh related EEG patterns (M1- and M2-related) that serve inverse functions.

Colocalization (M1/M2) modifies but does not alter the overall dominant signaling profile and a more apt description of the non-dominant receptor is subversive partner rather than outright antagonism. It is seen quite clearly in the striatum with dopamine receptor colocalizations i.e., D1/D3 and D2/D5. This doesn't deny additional convergent signaling factors that have delayed functional effects for the neuron.

### Laminar profile

ACh has a layer-specific effect in the rat primary somatosensory cortex (S1) (Eggermann and Feldmeyer, 2009). The near absolute hyperpolarization of layer 4 spiny stellate neurons (L4) to more generalized depolarizing responses of L2/3 and L5 pyramidal cells were predicted in Vakalopoulos (2006). Analogous findings were made in primary visual and auditory cortices and are mediated by K_ir_ channels. In the monkey visual cortex M2 heteroreceptor is selectively present in layers 4A and 4Cb and the interblob patches of L2/3 (Mrzljak et al., 1996). The parvocellular, but not koniocellular cells of the dorsolateral geniculate nucleus of the thalamus (dLGN) project to these layers. The koniocellular group project to cytochrome oxidase positive blobs of L2/3. An optogenetic study of the CA1 region of the mouse hippocampus revealed relative segregation of populations of interneurons that exhibited either M4R hyperpolarizations or likely M1-type (?M3, possibly M2) depolarizations (Bell et al., 2013).

Increasing cholinergic activation to neonatal rat visual cortex by basal forebrain stimulation generates discrete power peaks in the alpha and beta frequency ranges (Hanganu et al., 2007). Application of the muscarinic agonist carbachol to slices of neonatal rat parietal cortex caused similar maximal power in alpha and beta spectrum as measured by whole cell recordings and local field potentials (Kilb and Luhmann, 2003). In *in vitro* preparations of the intact cortex the power spectrum shifted from beta and low gamma to alpha at the end of the measured oscillatory cycle.

The overall findings corroborate the concept of M2 related gating of conscious visual information correlated with alpha power and an independent M1 activated pathway associated with underlying EEG gamma and or beta components that represents putative preattentive capacity.

### Caveats on alpha

The evidence for a muscarinic receptor-mediated mechanism generating alpha rhythms is compelling, but clear evidence for subtype specificity is lacking. Unlike for delta, M2 related GIRK activation in alpha, albeit plausible has not been directly examined nor has the role of M1R. Alpha spindles, induced by application of muscarinic agonist to thalamocortical networks, are blocked by pirenzipine and 4-DAMP, M1, and M3 antagonists, respectively (Lörincz et al., 2008). However, the antagonists are not entirely selective, pirenzipine being an inverse agonist (Daeffler et al., 1999) and 4-DAMP an antagonist at M2R (Collins et al., 1993) and cholinergic afferents are more closely associated with M2 rather than M3 (Oda et al., 2007). It also appears unlikely that the thalamus drives cortical alpha rhythms based on poor correlation (da Silva et al., 1973).
